# More twins expected in low-income countries with later maternal ages at birth and population growth

**DOI:** 10.1093/humrep/deae276

**Published:** 2024-12-26

**Authors:** D Susie Lee, Kieron J Barclay

**Affiliations:** Laboratory of Fertility and Well-Being, Max Planck Institute for Demographic Research, Rostock, Germany; Laboratory of Fertility and Well-Being, Max Planck Institute for Demographic Research, Rostock, Germany; Swedish Collegium for Advanced Study, Uppsala, Sweden; Department of Sociology, Stockholm University, Stockholm, Sweden

**Keywords:** twinning rate, maternal age structure, Demographic Health Surveys, UN World Population Prospects, sub-Saharan Africa, Southern Asia

## Abstract

**STUDY QUESTION:**

How are the changing maternal age structure and population growth expected to shape future twinning rates in low-income countries?

**SUMMARY ANSWER:**

With maternal age at birth projected to shift toward older ages, twinning rates are also estimated to increase in most low-income countries by 2050 and even more by 2100.

**WHAT IS KNOWN ALREADY:**

Many of the sub-Saharan African and South Asian countries are undergoing, and projected to further experience, the shift of maternal age at birth to older ages. Advanced maternal age is a well-established predictor of multiple births at the individual level, but currently, it is unknown how the changes in maternal age distribution are associated with the changes in twinning rates at the population level in low-income countries.

**STUDY DESIGN, SIZE, DURATION:**

We first estimated age-specific twinning probability based on Demographic Health Surveys and World Fertility Surveys data. We then scaled up the age-specific twinning probability at the population level to estimate changes in the number of twin births in 2050 and 2100 attributable to the estimated shifts in maternal age toward older ages as projected by the UN World Population Prospects (WPP).

**PARTICIPANTS/MATERIALS, SETTING, METHODS:**

We analyzed ∼3.19 million births that occurred within 10 years before the interview. Majority of the births in our data took place between 1980 and 2015 across 39 countries, where the uptake of medically assisted reproduction (MAR) is known to have been low during the observation period. We estimated country fixed-effects models to obtain country-specific twinning rates and age-specific twinning probability. We applied these estimates to the future number of births projected by the UN WPP, to estimate the number of twin births in 2050 and 2100.

**MAIN RESULTS AND THE ROLE OF CHANCE:**

With maternal age at birth projected to shift toward older ages, twinning rates are also estimated to increase in most countries by 2050 compared to 2010 (increases from 0.3% to 63% depending on countries), and even more in all studied countries by 2100 (increases from 3.5% to 79%). Due to its large population size, India will continue to have among the largest share of twin births despite its estimated decline of twin births by 10.5% by 2100. Nigeria, due to its not only large and growing population size but also high twinning rate, is expected to contribute the second largest number of twin births.

**LIMITATIONS, REASONS FOR CAUTION:**

Although the accuracy in maternal recall of multiple births tends to be high, our use of data based on recalled births from the past nonetheless imply a potential bias in our estimation of twinning rates.

**WIDER IMPLICATIONS OF THE FINDINGS:**

The present study suggests that, even if the spread of MAR remains slow in many low-income countries, twinning rates and number of twin births are expected to grow as an increase in maternal age at birth and population growth continue. Our findings call for more public health attention and societal support to be paid to twins and their families in low-income countries, given that twins are at higher risk of developmental challenges and health disadvantages.

**STUDY FUNDING/COMPETING INTEREST(S):**

D.S.L. was supported by the European Union (ERC, BIOSFER, 101071773). K.J.B. was supported by a Pro Futura Scientia XIV Fellowship awarded by the Swedish Collegium for Advanced Study and Riksbankens Jubileumsfond. There are no competing interests to declare.

**TRIAL REGISTRATION NUMBER:**

N/A.

## Introduction

The twinning rate, defined as the number of twins per 1000 deliveries, has increased nearly 10% during the past decades over the globe (Monden *et al.*, 2021). The variation in twinning rates is largely driven by the variation in dizygotic twinning rates, since monozygotic twinning rates are constantly low at 3–4 per 1000 deliveries across populations and that the vast majority of multiple births are in the form of twins versus higher-order multiple gestations (Beck *et al.*, 2021). The increase in twinning rates is intricately related to the global decline in fertility, because the shift in maternal age structure (the distribution of births by age of the mother) to older age, a key characteristic of fertility decline, is associated with a higher rate of dizygotic twinning (Bulmer, 1970). Many scholarly works from high-income countries have also pointed out the expansion of medically assisted reproduction (MAR) as the major reason for the increase in twinning rates (Pison *et al.*, 2015). Less is known about how twinning rates change in low-income countries (Smits and Monden, 2011; Monden *et al.*, 2021), where the literature indicates low availability and uptake of MAR due to financial and cultural barriers (Dyer *et al.*, 2020; Chiware *et al.*, 2021).

A fuller understanding of whether and to what extent twinning rates will increase in low-income countries is critical to assess the relative need for public health investment, for at least two reasons. First, multiple gestations are at greater risk of adverse perinatal outcomes as well as maternal morbidities (Vogel *et al.*, 2013; Monden and Smits, 2017), and children from multiple births are more prone to experience disadvantaged developmental trajectories (Sutcliffe and Derom, 2006). In sub-Saharan Africa, the twin-singleton gap in under-5 mortality has grown larger over the past decades, suggesting that twins have benefited less from the overall decline in early-life mortality (Monden and Smits, 2017). Second, there is great potential for the share and number of twins to increase in currently low-income countries. Sub-Saharan Africa exhibits the highest baseline twinning rate, at 16.8 as of 2010–2015, which, in combination with the high fertility rate, accounts for the region having the highest regional share (34%) of absolute number of twin deliveries in the world (Monden *et al.*, 2021). South Asia has the next highest share (19%) due to its large population size (Monden *et al.*, 2021). The share of global twin births by both regions will likely increase further, with the contribution of these regions to global population projected to increase in the coming decades (Department of Economic and Social Affairs, Population Division, 2022).

Independently of the use of MAR techniques like ovarian stimulation during *in vitro* fertilization (IVF), childbearing at older ages is associated with a higher probability of twinning, supported by robust evidence from various populations (Bulmer, 1970; Sear *et al.*, 2001; Pison *et al.*, 2015; Varella *et al.*, 2018; Rickard *et al.*, 2022). Humans and many other mammals overproduce zygotes, a strategy that has evolved to adjust optimal brood size in unpredictable environments and to selectively abort fetuses based on quality (Kozlowski and Stearns, 1989). Indeed, the majority of twin conceptions convert to singletons or are aborted entirely (vanishing twin syndrome), largely due to the high prevalence of structural and chromosomal anomalies in multiple conceptions (Landy and Keith, 1998). Even without significant defects, twin pregnancy imposes higher energetic demands and health risks on mothers (Santana *et al.*, 2016; Wierzejska, 2022). Thus, given the relative frailty and maternal costs associated with multiple gestations, frequency of twin births is expected to increase only if multiple gestations become less costly or even advantageous. Older maternal age could be one such condition due to the low remaining future reproductive chance.

Evolutionary theory suggests that when a female is near the end of her reproductive lifespan, carrying twins may represent an adaptive strategy that increases lifetime reproductive success even at the risk of carrying frail embryos such as offspring with genetic or epigenetic defects (Anderson, 1990). One proposed mechanism is that the physiological mechanisms related to fetal screening are relaxed at older maternal ages (Forbes, 1997). Growing evidence suggests that the human endometrium evolved as a biosensor of embryo quality (Macklon and Brosens, 2014). Although relaxing the ‘quality control’ would allow more frail embryos to implant, doing so may still be favored since any chemical signals of embryo frailty would be imperfect (Bruckner *et al.*, 2019). Increasing polyovulation at older ages is another mechanism believed to have evolved as a compensatory mechanism to counteract a higher risk of fertilization failure, embryo defects, and fetal loss when reproducing at old age (Anderson, 1990). The prevalence of multifollicular development increases with age (Beemsterboer *et al.*, 2006), and a simulation analysis has shown that switching to polyovulation at older ages could increase lifetime reproductive success (Hazel *et al.*, 2020). In sum, older mothers are more prone to multiple births, due to less stringent fetal screening and higher rates of polyovulation independently of the uptake of MAR.

It remains unclear to what extent the twinning propensity by maternal age, which is well-established at the individual level, underlies the changes in twinning rates at the *population* level (Elwood, 1985; Goswami and Goswami, 1993). Addressing this gap is important in low-income countries, where the ongoing decline in fertility is characterized by reproductive transitions at later ages (Bongaarts *et al.*, 2017; Pesando and GFC Team, 2019). Some of the currently low-income countries exhibit the highest twinning rates and large population size, already contributing the largest share to global twin births (Monden *et al.*, 2021). As such, the ongoing and projected population change in low-income countries implies more twins in the future and concomitantly additional health care costs associated with multiple births.

The present study seeks to better understand how the changing maternal age structure is expected to shape future twinning rates and number of twin births in low-income countries. To do so, we first estimate the relation between the proportion of women reproducing at older age and twinning rates at the population level, using the data from Demographic Health Surveys (DHS) and World Fertility Surveys (WFS) conducted in 39 low-income countries. We then project future changes in the rates and number of twin births for countries in which future population estimates are available from the UN World Population Prospects (WPP).

## Materials and methods

### The role of maternal age at birth in the past trends of twinning rates

#### Data

The DHS is a household survey based on a nationally representative sample of women aged 15–49 years old. The WFS program, the forerunner to the DHS, was conducted in low-income countries between 1974 and 1983. The analytic sample contains ∼3.19 million births, which occurred within 10 years from the time of interview from 162 DHS and WFS (Supplementary Table S1). The majority of the births took place in our data between 1980 and 2015 from ∼1.38 million mothers across 39 countries. To minimize the possibility that multiple births observed in the data are attributable to MAR, we excluded South Africa and all births after 2000 from India, based on the literature suggesting relatively active uptake of MAR in those countries (Malhotra *et al.*, 2013; Ombelet and Onofre, 2019). We compiled individual birth histories and restructured the data so that the unit of observation is a unique birth from each mother, paired with information on maternal age at birth (continuous years in 1-year intervals), parity (continuous integers), year of birth, and mother’s highest education. A birth can be either singleton or multiple, the latter of which are nearly 99% twins (Beck *et al.*, 2021). Although the DHS data do not distinguish monozygotic from dizygotic twins, the variation in twinning rates is largely driven by dizygotic twinning.

#### Probability of twinning by maternal age at childbirth

We first estimated the association between maternal age and twinning at the level of individual births. We used a linear probability model, which, compared to logistic regression model, produces predicted probabilities much closer to the observed distribution in cases of very rare binary events (Timoneda, 2021) such as twinning. We specified the model as:


(1) $${\mathrm{twin}\;\mathrm{birth}}_{i,c}=\;\beta {\mathrm{Maternal}\;\mathrm{age}\;\mathrm{at}\;\mathrm{childbirth}}_{i}+\gamma_{c}+\varepsilon_{i,c}$$

where ${\mathrm{twin}\;\mathrm{birth}}_{i,c}$ is a binary outcome of either twin or singleton, of a mother *i* from a country *c* in year *y*. β refer to the relationship between the outcome with maternal age at childbirth of the mother *i* at the given birth, categorized at 5-year intervals (15–19, 20–24, 25–29, 30–34, 35–39, 40–44, 45–49). We estimated maternal age at childbirth as dummies of 1- or 2-year intervals, and found very similar association with the probability of twinning (Supplementary Fig. S1). Since the numbers of women giving births projected by the WPP do not break further by parity, we opted to obtain maternal age estimates that would reflect the combined effects of maternal age and parity, two of which tend to positively correlate especially in high fertility contexts. Unlike for maternal age, evidence is mixed whether parity is positively associated with twinning (Bulmer, 1970; Bønnelykke, 1990). In the present data, positive correlations between parity (independently of age) and the probability of twinning appear to exist up around age 30 years, suggesting that higher-order births at older maternal ages might not necessarily increase twinning probabilities (Supplementary Table S2). In contrast, the probability of twinning increases independently of parity with maternal age before entering 40s, and the absolute values of coefficients are overall larger for maternal age. Taken together, we believe that the pattern of twinning by the combined maternal age and parity effects captured from the model will be mostly reflective of the maternal age effect.

The country fixed-effects $\gamma_{c}$ captures country variation in twinning rates that are allowed to differ by years *y*. The model thus effectively estimates how, on average, the probability of giving a twin birth differs by individuals within each country. Country fixed-effects were our preferred choice over mother fixed-effects (i.e. comparison of births within mothers) due to the well-known challenges when estimating individual fixed-effects for rare binary outcomes. One problem is the unstable and inflated coefficients, because baseline event risks are not reliably estimated for rare binary events. The problem is exacerbated because maternal age and measures of period changes are difficult to distinguish in maternal fixed-effects models (Holford, 2005). Moreover, due to the rarity of twinning, mothers who never delivered a twin (>96% of our sample) would be dropped in the maternal fixed-effects models. This would reduce the ecological validity of maternal age coefficients, because they are estimated based on the selected group of women who likely have a high propensity for twinning. With country fixed-effects, we retain mothers who have not delivered a twin birth, while still adjusting for time-invariant characteristics that differ between countries and may influence the probability of a twin delivery (e.g. genetic differences, average living conditions, and lifestyles and consumption patterns). We nevertheless estimate mother fixed-effects models for robustness checks (Supplementary Table S3). As another robustness check, we conducted a meta-analysis for the coefficients of maternal age categories estimated separately for each country (Supplementary Fig. S2).

#### Relationship between twinning rates and maternal age structure

To test if and to what extent maternal age structure explains period twinning rates at the country level, we created country panel data. The data were prepared first by grouping all births within a country by birth year. For births from too early or too recent years, for which the number of available observations is small, we combined adjacent birth years such that there are minimum 4000 deliveries observed within a group of birth years. The minimum was determined by simulations of the number of observations needed to ensure that at least 50 twin births are realized by chance alone given twinning rates between 15 and 20. This is to have enough twin births from which coefficients can be reliably estimated. Next, we calculated number of twins per 1000 deliveries for each birth year (or birth year groups) in a given country. As a result, we obtained an unbalanced panel sample of 35 countries for which enough births were observed. We removed countries (Central African Republic, The Gambia, Comoros, and Maldives) of which the observed births covered a period <10 years to increase the balance in the data structure. We estimated the following panel regression model:


(2) $${\mathrm{twinning}\;\mathrm{rate}}_{c,y}=\;\alpha +\beta_{1}\bar{{\mathrm{MAB}}_{c,y}}+\gamma_{c}+\theta_{y}+\varepsilon_{c,y}$$

Here, ${\mathrm{twinning}\;\mathrm{rate}}_{c,y}$ is the number of twin births per 1000 births, for country c in year y; $\gamma_{c}$ and $\theta_{y}$ are country and year fixed-effects respectively. $\bar{{\mathrm{MAB}}_{c,y}}$ is average maternal age at childbirth in country c in year y. Averages were categorized into ‘below 26’, ‘between 26 and 26.5’, ‘between 26.5 and 27’, and ‘above 27’, as the average maternal age at childbirth was concentrated in relatively narrow windows (mean = 26.3, interquartile range [IQR]: 25.8–26.9).

Equation (2) estimates the association between twinning rate and maternal age structure. The association is estimated from within-country variation through the country indicators $\gamma_{c}$, while controlling for shared time trends $\theta_{y}$. As such, the model seeks to get closer to the causal relationship between twinning rates and maternal age structure, albeit still associational. In separate models, we additionally controlled for period measures of gross domestic product (GDP) per capita and the proportion of women finishing at least primary education, to address the possibility that the association between twinning rates and maternal age structure is confounded by general improvement in living standards. GDP per capita is positively correlated with the utilization of IVF (Lass *et al.*, 2019), which increases the prevalence of multiple births independently of maternal age. Women with more education, through their better health conditions and access to public health care, are also more likely to give multiple births (Bhalotra and Clarke, 2019). We used whether or not a woman finishes at least primary education as the threshold for educational attainment, as research shows that potential gains in earnings increase substantially from secondary education (Wodon *et al.*, 2018).

Due to the relatively small number of observation points (period-country pairs), it was not feasible to relax the assumption of a shared time trend between countries by introducing country-specific time trends ($\gamma_{c}y$). We cannot therefore test if the estimates for $\beta_{1}$ are biased by time trends of certain countries. In an effort to partly mitigate the risk, we re-specified year fixed-effects as years grouped into 5-year intervals, thereby ‘smoothing’ out yearly variation across countries. Early and recent years for which observations are fewer were grouped into either ‘before 1980’ or ‘after 2015’.

### Estimating the number of future twin births

#### Scenarios and data

We took the following information from the WPP medium variant scenario (Department of Economic and Social Affairs, Population Division, 2022): The age distribution of women who gave births in the year 2010 (baseline age distribution), the age distribution of women who are projected to give birth in the years 2050 and 2100 (hypothetical age distribution), total number of births in the year 2010 (baseline population size), and total number of births projected for the years 2050 and 2100 (hypothetical population size).

#### Quantifying the number of future twin births

To estimate the number of twin births for different combinations of maternal age distribution and population size, we started from the notion that total number of births (b) is the sum of total number of deliveries (d) and total number of twin deliveries ($d_{t})$. Each delivery can be that of an either singleton or twin, assuming that all multiple births are twin births (Beck *et al.*, 2021). That is,


(3) $$b=d+\;d_{t}$$

Although b is usually known from vital statistics or projected estimates, d is not known. The value of d can be derived using b and baseline twinning rate (tr), in the following way. Using the fact that,


(4) $$tr=\;\frac{d_{t}}{d}$$

we can use (3) and (4) to solve for d, as follows.


(5) $$d=\frac{b}{\left(1+tr\right)}$$

**Figure 1.**
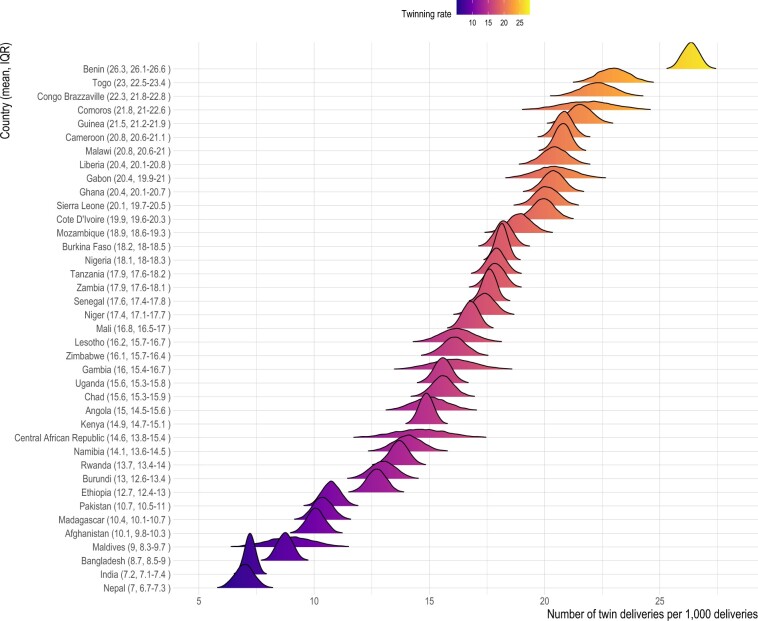
**Twinning rates estimated based on the Demographic Health Surveys data. Posterior distribution of country fixed-effects taken from a regression model.**  ${\mathrm{twin}\;\mathrm{birth}}_{i,c}=\;\beta {\mathrm{Maternal}\;\mathrm{age}\;\mathrm{at}\;\mathrm{childbirth}}_{i}+\gamma_{c}+\varepsilon_{i,c}$ where ${\mathrm{twin}\;\mathrm{birth}}_{i,c}$ is a binary outcome of either twin or singleton, of a mother i from a country c in year y. β refer to the relationship between the outcome with maternal age at childbirth of the mother i at the given birth, categorized at 5-year intervals (15–19, 20–24, 25–29, 30–34, 35–39, 40–44, 45–49). IQR, interquartile range (Q1–Q3).

To estimate the total number of deliveries (d) for a given year in a country using Equation (5), we used the number of births (b) taken from the WPP, and the posterior distribution of country-specific twinning rate (tr) obtained from the abovementioned Equation (1) only with country fixed-effects, i.e. average twinning rates for each country over the observed period. Given the higher variation in twinning rates between countries than within countries (Fig. 1), we believe that the tr estimates reflect baseline twinning rates that stay relatively constant over time within countries. Nonetheless, to the extent that the temporal fluctuations (be it structural or random) in country-specific twinning rates are captured in the DHS data, the posterior distributions allow us to incorporate uncertainties in the tr estimates and in turn in the estimates of d.

Once d is obtained, we partitioned the total number of deliveries into maternal age categories, using the maternal age structure (proportion of women giving births by age groups) available from the WPP. To the number of total deliveries for each age group, we multiplied the posterior distribution of age-specific twinning probability obtained from the regression model (Equation 1), to estimate the number of twin deliveries ($d_{t}$) for each age group in a country for different scenarios. For example, estimate for $d_{t}$ in 2100 for a given country can be obtained by applying b and maternal age structure of 2100, but the obtained $d_{t}$ will reflect the impact of changes in both population size and maternal age structure. To gauge the impact of changing maternal age structure on $d_{t}$, the projected maternal age structure of 2100 is applied while holding b at the level in 2010. As a metric for presenting projection results, we use the relative change (%) compared to 2010 rather than absolute number of twin births. In a Supplementary Table S5 and Fig. S4, we examined different sources of the projected changes in twinning rates and the number of twin births. For example, a shift in the maternal age structure toward older ages can occur via either an increase in the distribution of older age at childbearing via changes in age-specific fertility rate (ASFR) or an increase in the proportion of older women, or both. Changes in these sources could independently increase twinning rates. Increases in the number of twin births, on the other hand, can be affected by either an increasing population size or an increase in the proportion of older women, or both.

All data preparation, analyses, and visualization were done using the R 4.4.0 (R Core Team, 2021).

## Results

### The role of maternal age at birth in the past trends of twinning rates

**Figure 2.**
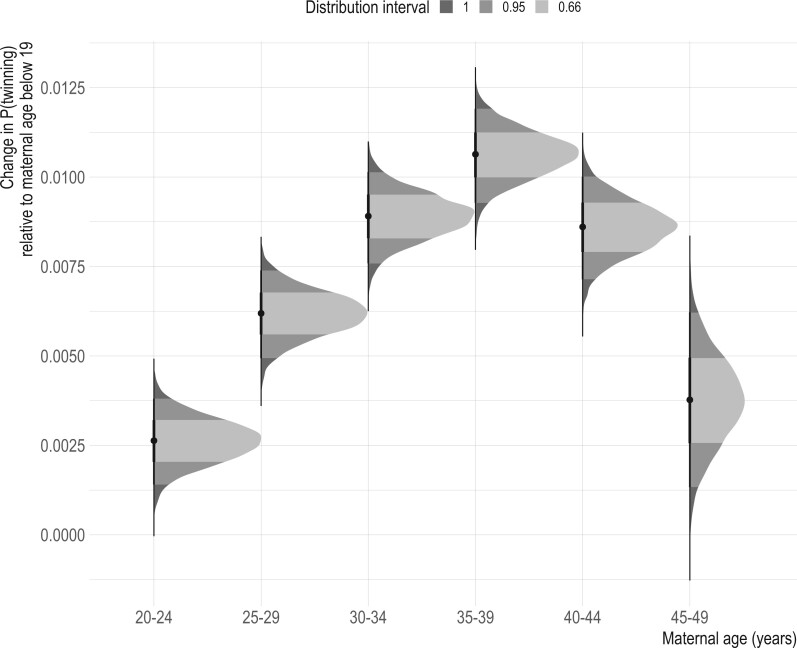
**Probability of twinning by maternal age at birth, relative to the probability at age 19 years and below.** Each distribution shows the posterior distribution of relative change in the probability of twinning, estimated based on the regression model ${\mathrm{twin}\;\mathrm{birth}}_{i,c}=\beta {\mathrm{Maternal}\;\mathrm{age}\;\mathrm{at}\;\mathrm{childbirth}}_{i}+\gamma_{c}+\varepsilon_{i,c}$ where ${\mathrm{twin}\;\mathrm{birth}}_{i,c}$ is a binary outcome of either twin or singleton, of a mother *i* from a country *c* in year *y*. β refer to the relationship between the outcome with maternal age at childbirth of the mother *i* at the given birth, categorized at 5-year intervals (15–19, 20–24, 25–29, 30–34, 35–39, 40–44, 45–49).

Average twinning rates varied across countries, ranging from 6.9 (IQR: 6.7, 7.3) in Nepal to 26.3 (IQR: 26.1, 26.6) in Benin (Fig. 1). After taking country differences into account, twinning is associated with maternal age at birth at the individual level. The probability that a woman delivers a twin birth increases with age, peaking at around 39 years old and only declining slightly afterward (Fig. 2). The age pattern in twinning is in general similar across the studied countries (Supplementary Fig. S2). Compared to the youngest age group, there is about 0.01 increase in the probability of twinning in late 30s (Fig. 2), and this amount of increase is similar to that reported from some high-income countries before the development of MAR (Pison *et al.*, 2015).

Note that the results presented above are based on individual-level analysis, where the variance to be explained is small to begin with (0.013) due to the rarity of twinning itself when examined across individuals. When the analysis is expanded to the country level in a panel regression, period twinning rates are higher when average maternal age at birth is older. Adjusting for country differences and shared time trends, period twinning rates are higher on average by 1.06 and 1.81 when average maternal age at birth on a given year in a country was 26.5–27 and above 27 years, respectively, compared to when average maternal age at birth was below 26 years (Table 1). The association between maternal age at birth and period twinning rates remained, after adding two potential factors—GDP per capita and proportion of women who finished at least primary education in a given year of a country—that could confound the association between period twinning rates and maternal age structure (Table 1).

**Table 1.** Regression coefficients^1^ of period twinning rates, for 35 countries from which yearly GDP information was available.

	*Dependent variable:*		
	Period twinning rate		
	(1)	(2)	(3)
Maternal age at birth: 26–26.5	0.452	0.637	0.708
	(0.450)	(0.469)	(0.481)
Maternal age at birth: 26.5–27	1.064*	1.356*	1.484*
	(0.526)	(0.549	(0.583)
Maternal age at birth: 27+	1.801**	2.041**	2.192**
	(0.649)	(0.663)	(0.703)
GDP per capita		0.000	0.000
		(0.000)	(0.000)
Proportion of women having at least primary education			1.334
			(2.018)
Observations	236	231	231
*R* ^2^	0.874	0.877	0.878
Residual SE	1.97 (df = 190)	1.97 (df = 184)	1.97 (df = 183)
F statistic	29.39 (df = 45; 190)	28.69 (df = 46; 194)	28 (df = 47; 183)

* *P* < 0.05.

** *P* < 0.01.

1 Panel regression was based on the formula ${\mathrm{twinning}\;\mathrm{rate}}_{c,y}=\alpha +\beta_{1}\bar{{\mathrm{MAB}}_{c,y}}+\gamma_{c}+\theta_{y}+\varepsilon_{c,y}$ where ${\mathrm{twinning}\;\mathrm{rate}}_{c,y}$ is the number of twin births per 1000 births, for country c in year y; $\gamma_{c}$ and $\theta_{y}$ are country and year fixed-effects respectively. $\bar{{\mathrm{MAB}}_{c,y}}$ is average maternal age at childbirth in country c in year y.

### Estimates of future twin births

#### Twinning rates

**Figure 3.**
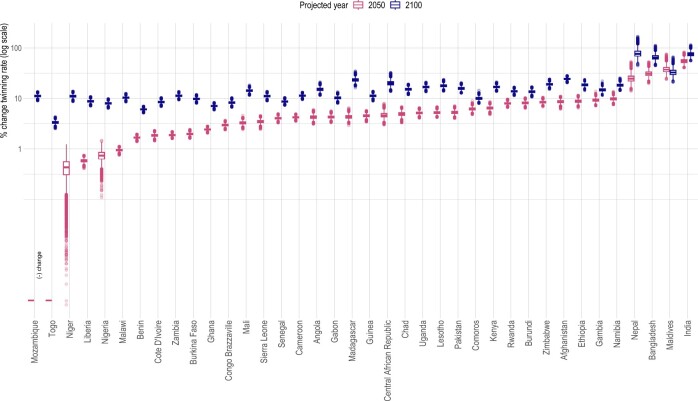
**Predicted percent change in twinning rates compared to 2010.** Values used to make the figure can be found in Supplementary Table S4.

With the distribution of births by the age of the mother (i.e. maternal age structure) projected to shift toward older ages (Supplementary Fig. S3), twinning rates are also likely to increase in most countries by 2050 compared to 2010 (increases from 0.4% to 55%), and even more in all studied countries by 2100 (increases from 3.4% to 79%) (Fig. 3, Supplementary Table S4). Exceptions for 2050 are Mozambique and Togo, where maternal age structure is projected to shift slightly toward younger age in 2050 compared to 2010, and consequently, twinning rates are expected to decline (red bars, Fig. 3). This drop in the twinning rates is very small (on average by 1% in both countries) and expected to reverse as maternal age structure begins to shift toward older age in these countries by 2100. Different modes by which maternal age structure changes in countries mean that twinning rates increase at different paces, as also illustrated by the relatively larger increase in the twinning rate estimated for Mozambique compared to Togo in 2100 (Fig. 3) due to the larger shift to in maternal age structure in Mozambique by 2100. By 2100, all studied countries are expected to have higher twinning rates than 2010, with the estimated increase by a minimum of 3.4% (Togo, IQR: 3.2–3.5) and a maximum of 79% (Nepal, IQR: 69–86) (blue bars, Fig. 3). We further find that an increasing ASFR at older ages and an increasing proportion of older women are predicted to contribute similar degree to the increasing rate of twinning, except in some Southern Asian countries, where the contribution of increasing ASFR is slightly larger (Supplementary Table S5 and Fig. S4). These countries—Bangladesh, India, and Nepal—are also predicted to show the largest increase in twinning rates. Put in another metric, the increase in twinning rates means that more children have a twin sibling in the future. In Nepal, for example, 1 out of every 48 children in Nepal is expected to have a twin sibling by 2050, compared to 1 out of every 72 in 2010. The number is reduced to 1 out of every 40 children in Nepal with a twin sibling by 2100.

#### Number of twin births

The absolute number of twin births is expected to increase in most but not all studied countries by 2100 compared to 2010 (Supplementary Table S4). In sub-Saharan Africa, with its population size projected to grow by 2100, the number of twin births is estimated to grow in most countries, from an increase of 10.6% in Kenya (IQR: 10.1–11.2) to 283% in Niger (IQR: 282–285). The same explanation applies for the continued increase in the number of twin births in some South Asian countries, such as Pakistan and Afghanistan. The predicted increase in the number of twin births is largely driven by the increasing population size than by the aging of maternal age structure (Supplementary Table S5 and Fig. S4), in most countries that are projected to experience further population growth by 2100. On the other hand, in Bangladesh and India, where fertility was already below replacement level in 2010, the number of women giving birth in the future is projected to be smaller. Consequently, the number of twin births is estimated to decline by 10.7% in India (IQR: 8.3–13.5) to and by 20.2% in Bangladesh (IQR: 17.9–22.8), as well as other much smaller Southern Asia countries such as Nepal and Maldives. The decline would be even larger (up to 50% instead of the 10–20%) if the age structure of women giving birth retained the pattern in 2010, i.e. if women giving birth were on average younger than would be expected for 2100. This suggests that more women giving birth at older ages, a key demographic trend accompanying the fertility decline, may ‘buffer’ the impact of declining population size on the number of twin births given the age-related increase in twinning propensity.

**Figure 4.**
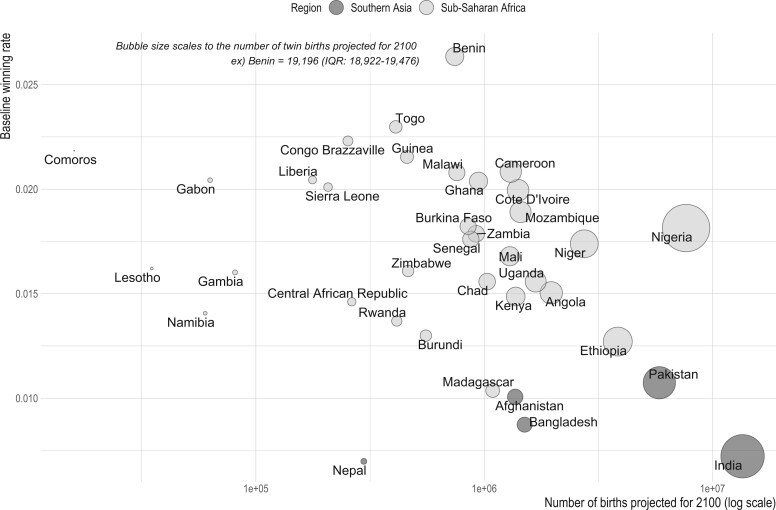
**Bubble chart showing the projected number of twin births for 2100, by the projected number of births (as proxy of population growth) and baseline twinning rates estimated from the Demographic Health Surveys (also see**  **Fig. 1****).**

Nonetheless, due to its large population size, India will continue to have among the largest share of twin births (23.4%) among the studied countries, despite its estimated decline of twin births by 10.5% by 2100. Among sub-Saharan African countries, Nigeria, due to its not only large and growing population size but also high baseline twinning rate (0.018), is expected to contribute the second largest number of twin births (13.2%) next to India (Fig. 4). Pakistan is expected to out-twin Ethiopia and Niger by 1.2–13 times in the number of twin births, despite its lower twinning rates and a smaller anticipated increase in the number of births compared to Ethiopia or Niger; it is Pakistan’s large population size that explains the larger share of twin births in Pakistan relative to Ethiopia and Niger.

## Discussion

The recent increase in twinning rates in high-income countries has been attributed largely to the increasing uptake of MAR that involve ovarian stimulation, but also to some degree higher mean ages at childbearing (Pison *et al.*, 2015; Monden *et al.*, 2021). The present study suggests that, even if the spread of MAR remains slow in many low-income countries, twinning rates are expected to grow as maternal age structure shifts toward older ages. In many countries, the degree of increase will be the same or even larger than the 40–50%, the average increase in twinning rates estimated from some high-income countries and attributed primarily to MAR (Pison *et al.*, 2015).

Larger increases in twinning rates are expected for South Asian countries with the largest expected increases in the proportion of births to older women. However, due to their low baseline twinning rates (Fig. 1), the expected future twinning rates for these South Asian countries will approximate the lower end of the twinning rates distribution for Sub-Saharan African countries. Nonetheless, the increase in twinning rates means that the relative increase experienced by these South Asian countries in terms of the need for supporting twin pregnancies and twin families will be significant. For example, our estimation suggests that delayed childbearing alone would result in 1 out of every 40 children in Nepal having a twin sibling by 2100, compared to 1 out of every 72 in 2010. The same comparison for Niger, on the other hand, is estimated to be 1 out of 29 in 2100 versus 1 out of 26 in 2010, due to the relatively slower shift in maternal age structure projected for this country. Instead, Niger, and other Sub-Saharan countries, are estimated to have more twin births in absolute number due to their projected continuation of population growth and already high twinning rates, although the increase in twinning rates is expected to remain slower than Southern Asian countries.

The increase in twinning rates is likely to be even larger, if we consider the expansion of other contributing factors not incorporated into our estimations. For example, the uptake of MAR has increased in India over the last two decades (Malhotra *et al.*, 2013), and there are ongoing efforts to make MAR more available and accessible in Africa (Ombelet, 2011; Ombelet and Onofre, 2019). To our best knowledge, there is no published estimate for the proportion of future births expected to be conceived through MAR. One study, based on data from some high-income countries during 1970–2005, reported an estimate of an average 40–50% increase in twinning rates if only the expansion of MAR had been in play, independently of delayed childbearing (Pison *et al.*, 2015). General improvement in population health is another factor that over time is likely to increase twinning rates (Eriksson and Fellman, 2004). A recent study finds that indicators of better maternal health or prenatal environment are positively associated with the probability of twinning in both low- and high-income countries, independent of maternal age at birth (Bhalotra and Clarke, 2019). Those indicators may refer to downstream mechanisms by which the improved maternal health and access to health care contribute to the chance of carrying a twin pregnancy to term. It remains to be seen how the numerous aspects of population health, separately or collectively, contribute to future twinning rates as improvements in living standards continue apace in low-income countries.

Our findings call for more public health attention to be directed toward twin births in many of the currently low-income countries. Early life survival is still lower among multiples compared to singletons, especially in the first year of life (Monden and Smits, 2017; Pongou *et al.*, 2019). Thus, although our study implies more twins will be *born*, an important task remains to ensure that these twins can *survive* their early life as often as singletons do. The twin-singleton gap is also present in terms of higher maternal mortality and morbidity associated with twin pregnancy (Vogel *et al.*, 2013; Santana *et al.*, 2016). To close these gaps, policies and guidelines for diagnosing and caring for twin pregnancies are critical (Hanson *et al.*, 2019). In addition to improving health care systems, improving household socioeconomic status could further reduce the survival disadvantage for twins. For example, ensuring educational opportunities for women could be one key factor, given the educational gradient in the survival penalty for twins: twins born to women with primary education or less are even more likely to die than those born to women with secondary education (Guo and Grummer-Strawn, 1993). Beyond survival, twins may have unique developmental needs that require further attention from both a health and educational policy perspective (Hay and Preedy, 2006).

While the age-dependent twinning propensity has long been known, our study expands the literature by explicitly considering its population-level consequences given the expected trend of delayed childbearing in low-income countries. Our approach could be further generalized, for example, to explain why twinning rates in some European countries fell between the 1950s and 1970s (Pison *et al.*, 2015), by considering the fact that mean maternal age at birth also declined during the same period (MacGillivray, 1981). With the large sample of births observed across many countries, we could reliably model the individual probability of twinning and derive the age-specific probability of twinning for projections. The age-specific pattern of twinning probability was largely consistent across the studied countries (Supplementary Fig. S2) and with previous studies that have demonstrated higher twinning rates amongst older mothers in both pre-industrialized and modern populations (Bulmer, 1970; Sear *et al.*, 2001; Pison *et al.*, 2015; Varella *et al.*, 2018; Rickard *et al.*, 2022).

The data used nonetheless have some limitations. First, even with the large sample size of DHS, some countries with low baseline twinning rate or small population size offered small numbers of observations for estimating age-specific twinning rates. The present study thus used data pooled across countries to estimate age-specific twinning probabilities. Although this may mask between-country variation, which remains to be examined in more detail in the future, we believe that the degree of such variation would be very small due to the generally low twinning rates. Second, recalled births from the past imply a potential bias in our estimation of twinning rates over time, if births that occurred in the past suffer from poor recall. Evidence suggests that the accuracy in maternal recall of multiple births is known to be high, comparable to cesarean section, according to a study on more than 22-year follow-up (Buka *et al.*, 2004), in part because multiple births are often given cultural salience (Pison, 1992) across time and space. Although this leads us to believe that past twin births are unlikely to be remembered less, we restricted our analysis to births occurred within 10 years preceding the surveys. Third, with the currently available data, the challenge of pinpointing a baseline twinning rate for a given country remains. In the absence of official registers for multiple births in the studied countries, we cannot assess to what extent the estimated country fixed-effects accurately capture country-specific baseline twinning rates. Previously reported measures may not provide useful reference points, since they are predominantly based on hospital or health care-based records, which tend to oversample multiple births (Bjerregaard-Andersen *et al.*, 2012). Previous reports also draw data from a particular region of a country, and their results are influenced by regional variation in twinning rates, e.g. from 8.5 to 46.5 in Nigeria (Akinseye *et al.*, 2019). The regional variation is likely real and substantial (Pison, 1992), but may also be partly due to sampling errors stemming from the low prevalence of twinning rates to begin with. Finally, twinning rates change over time even during the pre-MAR period (Elwood, 1985; Goswami and Goswami, 1993), making it even more problematic to assume a baseline twinning rate for a given country. Taking these considerations together, we presented our main projection results (Fig. 3) in terms of relative change (%) in the rate and number of twin births rather than absolute value of changes (e.g. number of excess twin births expected in future), while presenting absolute number of twin births only in relation to the estimated twinning rates (Fig. 4) for interested readers. We also note that the baseline twinning rates estimated from the DHS broadly replicate the well-known high twinning rates in Sub-Saharan Africa compared to Asia (Bulmer, 1970; Pison, 1992).

To conclude, the present study examines how the twinning propensity by maternal age may underlie the changes in twinning rates at population level, in the context of low availability and uptake of MAR. The projected increase in twinning rates should reflect a minimum level, because the rate would increase even more if MAR becomes more prevalent as it has in high-income countries. The present study also highlights the expected increase in the number of twin births associated with population growth. Thus, more attention is required in the future to support healthy outcomes for twin pregnancies and families with twins.

## Supplementary Material

deae276_Supplementary_Figure_S1

deae276_Supplementary_Figure_S2

deae276_Supplementary_Figure_S3

deae276_Supplementary_Figure_S4

deae276_Supplementary_Table_S1

deae276_Supplementary_Table_S2

deae276_Supplementary_Table_S3

deae276_Supplementary_Table_S4

deae276_Supplementary_Table_S5

## Data Availability

The original data used for the analyses are available on the Internet free of charge. Demographic and Health Surveys data are available for download for registered users from https://dhsprogram.com/. The World Population Prospects 2022 data are downloadable from the United Nations Population Division website https://population.un.org/wpp/. All R codes for conducting the analyses presented in this paper are available at https://github.com/DSusieLee/twinage.
