# Supplementary figures and images for: More twins expected in low-income countries with later maternal ages at birth and population growth
Source: Hum Reprod. 2024 Dec 26;40(2):372–81. doi: 10.1093/humrep/deae276 (PMC11788213; doi:10.1093/humrep/deae276)

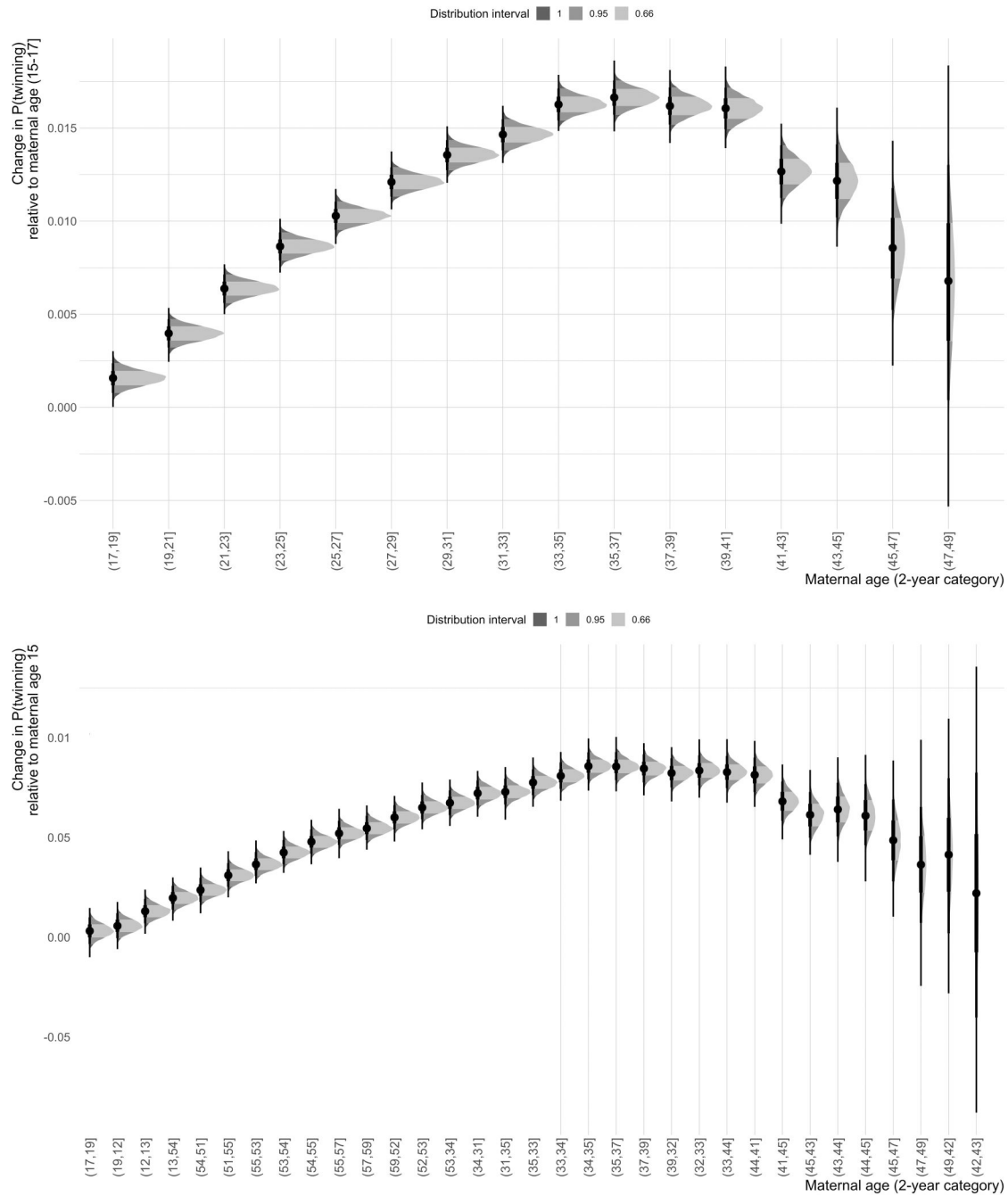

Supplement: deae276_Supplementary_Figure_S1 [file deae276_supplementary_figure_s1.pdf]

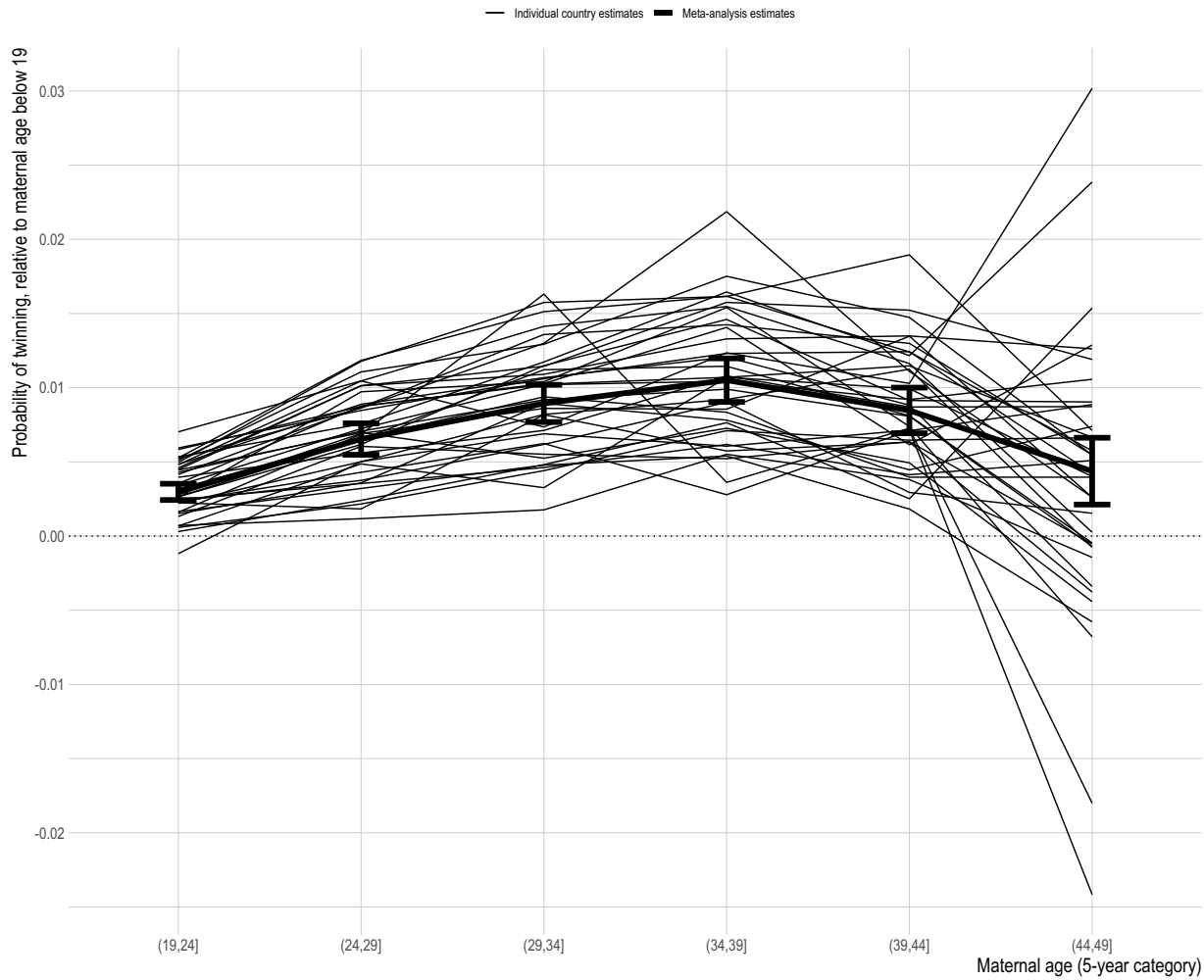

Supplementary Figure S2. Meta-analysis of maternal age coefficients.

Supplement: deae276_Supplementary_Figure_S2 [file deae276_supplementary_figure_s2.pdf]
