# Supplementary material for: More twins expected in low-income countries with later maternal ages at birth and population growth
Source: Hum Reprod. 2024 Dec 26;40(2):372–81. doi: 10.1093/humrep/deae276 (PMC11788213; doi:10.1093/humrep/deae276)
Supplement: deae276_Supplementary_Figure_S4 [file deae276_supplementary_figure_s4.pdf]

OXFORD

human reproduction

Human Reproduction, 2024, 1–1
https://doi.org/10.1093/humrep/deae276
Supplementary Data

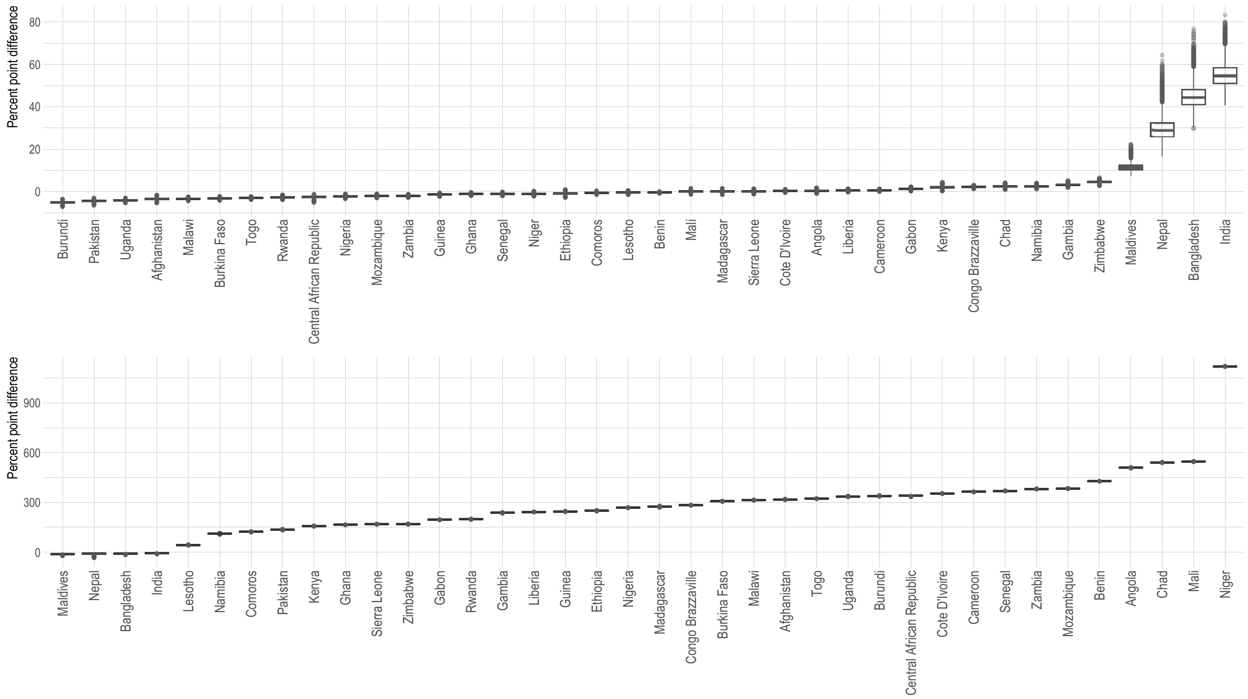

Supplementary Figure S4. Percent point difference between the predicted percent changes in twinning rates (top) and number of twin births (bottom).
