# Supplementary material for: More twins expected in low-income countries with later maternal ages at birth and population growth
Source: Hum Reprod. 2024 Dec 26;40(2):372–81. doi: 10.1093/humrep/deae276 (PMC11788213; doi:10.1093/humrep/deae276)
Supplement: deae276_Supplementary_Table_S1 [file deae276_supplementary_table_s1.pdf]

Human Reproduction, 2024, 1–1
https://doi.org/10.1093/humrep/deae276
Supplementary Data

Supplementary Table S1. Number (N) of live births recorded in Demographic Health Surveys (DHS) and World Fertility Survey (WFS) used in the study.

Country	Year	N	Country	Year	N	Country	Year	N	Country	Year	N
Afghanistan	2015	66529	Ethiopia	2000	21877	Malawi	1992	9041	Senegal	1978	7879
Afghanistan	2016	4044	Ethiopia	2005	20888	Malawi	2000	22610	Senegal	1986	8526
Angola	2015	13543	Ethiopia	2010	2185	Malawi	2004	15866	Senegal	1992	3793
Angola	2016	12261	Ethiopia	2011	21563	Malawi	2005	4668	Senegal	1993	7424
Bangladesh	1993	6462	Ethiopia	2016	21798	Malawi	2010	39968	Senegal	1997	14567
Bangladesh	1994	9141	Gabon	2000	8649	Malawi	2015	25336	Senegal	2005	20937
Bangladesh	1996	7194	Gabon	2001	125	Malawi	2016	10232	Senegal	2010	9213
Bangladesh	1997	6231	Gabon	2012	10976	Maldives	2009	7351	Senegal	2011	14187
Bangladesh	1999	6407	Gambia	2013	14857	Maldives	2016	1343	Senegal	2012	4969
Bangladesh	2000	7538	Ghana	1979	10130	Maldives	2017	5214	Senegal	2013	8003
Bangladesh	2004	13874	Ghana	1980	274	Mali	1987	6816	Senegal	2014	12847
Bangladesh	2007	12858	Ghana	1988	7751	Mali	1995	7032	Senegal	2015	13532
Bangladesh	2011	19346	Ghana	1993	7337	Mali	1996	13544	Senegal	2016	26352
Bangladesh	2014	17480	Ghana	1994	312	Mali	2001	25652	Senegal	2017	24604
Bangladesh	2017	9931	Ghana	1998	3685	Mali	2006	28933	Sierra Leone	2008	11836
Bangladesh	2018	8235	Ghana	1999	3072	Mali	2012	13309	Sierra Leone	2013	25407
Benin	1981	90	Ghana	2003	7848	Mali	2013	6998	Sierra Leone	2019	20688
Benin	1982	7831	Ghana	2008	6145	Mali	2018	19774	South Africa	1998	10533
Benin	1996	10282	Ghana	2014	12042	Mozambique	1997	13698	South Africa	2016	7243
Benin	2001	10753	Ghana	2019	932	Mozambique	2003	20266	Tanzania	1991	9068
Benin	2006	32279	Ghana	2020	5144	Mozambique	2004	177	Tanzania	1992	6463
Benin	2011	2223	Ghana	2021	5918	Mozambique	2011	21089	Tanzania	1996	13597
Benin	2012	23674	Guinea	1999	12524	Namibia	1992	7528	Tanzania	1999	6425
Benin	2017	13471	Guinea	2005	13883	Namibia	2000	8275	Tanzania	2004	11596
Benin	2018	12244	Guinea	2012	14670	Namibia	2006	2808	Tanzania	2005	4824
Burkina Faso	1992	2211	Guinea	2018	16098	Namibia	2007	7138	Tanzania	2009	1381
Burkina Faso	1993	9200	India	1992	53861	Namibia	2013	9657	Tanzania	2010	13847
Burkina Faso	1998	3806	India	1993	77216	Nepal	1976	11350	Tanzania	2015	14718
Burkina Faso	1999	8044	India	1998	20550	Nepal	1996	14609	Tanzania	2016	5137
Burkina Faso	2003	22427	India	1999	100355	Nepal	2001	14272	Tanzania	2022	20591
Burkina Faso	2010	31430	India	2000	1294	Nepal	2006	12270	Togo	1988	6135
Burkina Faso	2021	25767	India	2005	8030	Nepal	2011	11437	Togo	1998	13997
Burundi	1987	7348	India	2006	103160	Nepal	2016	10794	Togo	2013	5366
Burundi	2010	13554	India	2015	281356	Nepal	2017	273	Togo	2014	8715
Burundi	2011	1191	India	2016	269527	Nepal	2021	898	Uganda	1988	7272
Burundi	2016	15957	Kenya	1977	8831	Nepal	2022	10121	Uganda	1989	1985
Burundi	2017	10393	Kenya	1978	8551	Niger	1992	13501	Uganda	1995	13231
Cameroon	1978	13648	Kenya	1988	943	Niger	1998	15395	Uganda	2000	6629
Cameroon	1991	6347	Kenya	1989	12614	Niger	2006	18676	Uganda	2001	6794
Cameroon	1998	7864	Kenya	1993	12485	Niger	2012	25781	Uganda	2006	16548
Cameroon	2004	15312	Kenya	1998	11346	Nigeria	1981	7703	Uganda	2011	15560
Cameroon	2011	22011	Kenya	2003	11225	Nigeria	1982	30	Uganda	2016	31176
Cameroon	2018	38236	Kenya	2008	5448	Nigeria	1990	16331	Zambia	1992	11694
Cameroon	2019	134	Kenya	2009	6331	Nigeria	2003	11688	Zambia	1996	13581
Central African Republic	1994	6060	Kenya	2014	44444	Nigeria	2008	57770	Zambia	1997	76
Central African Republic	1995	3476	Kenya	2022	38202	Nigeria	2013	62067	Zambia	2001	2865
Chad	1996	1675	Lesotho	1977	6685	Nigeria	2018	70198	Zambia	2002	10091
Chad	1997	12581	Lesotho	2004	6738	Pakistan	1975	11344	Zambia	2007	11999
Chad	2004	11699	Lesotho	2005	566	Pakistan	1990	3007	Zambia	2013	16517
Chad	2014	13089	Lesotho	2009	6546	Pakistan	1991	11964	Zambia	2014	10338
Chad	2015	25042	Lesotho	2010	991	Pakistan	2006	14406	Zambia	2018	19971
Comoros	1996	4138	Lesotho	2014	6345	Pakistan	2007	5599	Zambia	2019	486
Comoros	2012	6413	Liberia	1986	9851	Pakistan	2012	18370	Zimbabwe	1988	5991
Congo Brazzaville	2005	9064	Liberia	2006	945	Pakistan	2013	6858	Zimbabwe	1989	832
Congo Brazzaville	2011	15149	Liberia	2007	10067	Pakistan	2017	8104	Zimbabwe	1994	8602
Congo Brazzaville	2012	2452	Liberia	2013	15519	Pakistan	2018	17970	Zimbabwe	1999	7265
Cote D'Ivoire	1980	7125	Liberia	2019	6761	Rwanda	1992	11551	Zimbabwe	2005	7873
Cote D'Ivoire	1981	4320	Liberia	2020	4986	Rwanda	2000	15670	Zimbabwe	2006	2277
Cote D'Ivoire	1994	14230	Madagascar	1992	10299	Rwanda	2005	16881	Zimbabwe	2010	5742
Cote D'Ivoire	1998	1989	Madagascar	1997	11893	Rwanda	2010	10504	Zimbabwe	2011	4587
Cote D'Ivoire	1999	2003	Madagascar	2003	3839	Rwanda	2011	7706	Zimbabwe	2015	11706
Cote D'Ivoire	2011	1309	Madagascar	2004	7102	Rwanda	2014	6151			
Cote D'Ivoire	2012	13530	Madagascar	2008	5293	Rwanda	2015	10072			
Cote D'Ivoire	2021	21869	Madagascar	2009	20045	Rwanda	2019	4442			
			Madagascar	2021	24015	Rwanda	2020	11237			
