# Supplementary material for: More twins expected in low-income countries with later maternal ages at birth and population growth
Source: Hum Reprod. 2024 Dec 26;40(2):372–81. doi: 10.1093/humrep/deae276 (PMC11788213; doi:10.1093/humrep/deae276)
Supplement: deae276_Supplementary_Table_S2 [file deae276_supplementary_table_s2.pdf]

OXFORD
human reproduction
Human Reproduction, 2024, 1–1
https://doi.org/10.1093/humrep/deae276
Supplementary Data

Supplementary Table S2. Maternal age effects on twinning by birth order (parity).

	Only maternal age at birth (MAB)		Maternal age at birth and parity		Maternal age at birth and parity interact	
MAB 19–23	0.005***	0.000	0.004***	0.000	0.004***	0.000
MAB 24–28	0.010***	0.000	0.007***	0.000	0.009***	0.000
MAB 29–33	0.013***	0.000	0.010***	0.000	0.014***	0.001
MAB 34–38	0.015***	0.000	0.012***	0.000	0.014***	0.002
MAB 39–43	0.014***	0.000	0.010***	0.000	0.013**	0.005
MAB 44+	0.009***	0.001	0.005***	0.001	0.026*	0.016
Parity 2			0.002***	0.000	0.003***	0.000
Parity 3			0.003***	0.000	0.006***	0.001
Parity 4			0.003***	0.000	0.005**	0.002
Parity 5+			0.005***	0.000	0.007	0.005
19–23 × Parity 2					−0.002***	0.001
24–28 × Parity 2					−0.002***	0.001
29–33 × Parity 2					−0.006***	0.001
34–38 × Parity 2					−0.004**	0.002
39–43 × Parity 2					−0.012**	0.006
44+ × Parity 2					−0.019	0.020
19–23 × Parity 3					−0.003**	0.001
24–28 × Parity 3					−0.004***	0.001
29–33 × Parity 3					−0.006***	0.001
34–38 × Parity 3					−0.005**	0.002
39–43 × Parity 3					−0.002	0.005
44+ × Parity 3					−0.022	0.018
19–23 × Parity 4					−0.001	0.002
24–28 × Parity 4					−0.004	0.002
29–33 × Parity 4					−0.005**	0.002
34–38 × Parity 4					−0.004	0.003
39–43 × Parity 4					−0.002	0.005
44+ × Parity 5+					−0.029*	0.017
19–23 × Parity 5+					0.001	0.005
24–28 × Parity 5+					−0.003	0.005
29–33 × Parity 5+					−0.006	0.005
34–38 × Parity 5+					−0.003	0.006
39–43 × Parity 5+					−0.005	0.007
44+ × Parity 5+					−0.023	0.017

Linear probability models with country fixed-effects were estimated to compare models where maternal age at birth (mab_cat) does not interact with parity and a model where they interact. Each coefficient quantifies the chance of twinning, and statistical significance is shown with asterisks (* for $P < 0.05$, ** for $P < 0.01$, *** for $P < 0.001$).
