# Supplementary material for: More twins expected in low-income countries with later maternal ages at birth and population growth
Source: Hum Reprod. 2024 Dec 26;40(2):372–81. doi: 10.1093/humrep/deae276 (PMC11788213; doi:10.1093/humrep/deae276)
Supplement: deae276_Supplementary_Table_S3 [file deae276_supplementary_table_s3.pdf]

OXFORD
human reproduction
Human Reproduction, 2024, 1–1
https://doi.org/10.1093/humrep/deae276
Supplementary Data

Supplementary Table S3. Mother fixed-effects results.

Model:	Mother fixed-effects		Country fixed-effects	
	**Maternal age only**	**Parity added**	**Maternal age only**	**Parity added**
	**Coefficient (SE)**			
Age: 20–24	0.0055*** (0.0003)	0.0098*** (0.0004)	0.0048*** (0.0004)	0.0036*** (0.0003)
Age: 25–29	0.0108*** (0.0003)	0.0231*** (0.0006)	0.0097*** (0.0009)	0.0071*** (0.0007)
Age: 30–34	0.0154*** (0.0005)	0.0347*** (0.0008)	0.0133*** (0.0011)	0.0098*** (0.0008)
Age: 35–39	0.0195*** (0.0006)	0.0422*** (0.0009)	0.0155*** (0.0011)	0.0116*** (0.0008)
Age: 40–44	0.0200*** (0.0009)	0.0444*** (0.0011)	0.0137*** (0.0011)	0.0095*** (0.0008)
Age: 45–49	0.0158*** (0.0018)	0.0412*** (0.0019)	0.0089*** (0.0013)	0.0047*** (0.0011)
Parity 2		0.0003 (0.0003)		0.0017*** (0.0004)
Parity 3		−0.0052*** (0.0004)		0.0032*** (0.0006)
Parity 4		−0.0120*** (0.0006)		0.0035*** (0.0006)
Parity 5+		−0.0238*** (0.0008)		0.0049*** (0.0007)
Adj. R^2	0.030	0.032	0.003	0.003

*** Indicates *P*-value <0.001.
Here, we present results from the mother fixed-effects models and compare maternal age estimates with those produced from country fixed-effects models. The models were estimated using the ‘feols’ function available from the R package ‘fixest’ (Bergé 2018). (Estimating mother fixed-effects with over 1 million mothers was not feasible with the Bayesian modeling.) The main takeaway from the results is that there is overall congruence in the pattern by maternal age categories. That is, the probability of twinning gradually increases up to late 30s and declines during 45–49 (but still higher than the probability for maternal age 15–19 years). Whether the probability begins to decline during early 40s or later is unclear. One important difference between mother fixed-effects and country fixed-effects is the impact of adding parity into the models on maternal age coefficients, especially the inflated maternal age coefficient estimates in the mother fixed-effects model. We observe the same if maternal age is categorized at 2-year intervals (results not shown here). One possible explanation is that the maternal age estimates ‘absorb’ some of the effects of time variables that change linearly together with maternal age. Based on the well-known challenge of distinguishing variables that scale with maternal age using mother fixed-effects model, we refrain from interpreting the parity coefficients. Given the unstable estimates from mother fixed-effects models, we prefer to use maternal age coefficients estimated from the country fixed-effects models.
