# Supplementary material for: More twins expected in low-income countries with later maternal ages at birth and population growth
Source: Hum Reprod. 2024 Dec 26;40(2):372–81. doi: 10.1093/humrep/deae276 (PMC11788213; doi:10.1093/humrep/deae276)
Supplement: deae276_Supplementary_Table_S4 [file deae276_supplementary_table_s4.pdf]

OXFORD human reproduction

Human Reproduction, 2024, 1–2
https://doi.org/10.1093/humrep/deae276
Supplementary Data

Supplementary Table S4. Projected percent change in twinning rates and number of twins compared to 2010.

Country	Scenario	Median	Mean	Q25	Q75
Mozambique	Twinning rates 2050	−0.010	−0.010	−0.011	−0.008
Togo	Twinning rates 2050	−0.009	−0.009	−0.010	−0.009
Niger	Twinning rates 2050	0.004	0.004	0.003	0.006
Liberia	Twinning rates 2050	0.006	0.006	0.006	0.006
Nigeria	Twinning rates 2050	0.007	0.007	0.006	0.009
Malawi	Twinning rates 2050	0.009	0.009	0.009	0.010
Benin	Twinning rates 2050	0.017	0.017	0.016	0.017
Cote D'Ivoire	Twinning rates 2050	0.019	0.019	0.018	0.019
Zambia	Twinning rates 2050	0.019	0.019	0.018	0.019
Burkina Faso	Twinning rates 2050	0.020	0.020	0.019	0.020
Ghana	Twinning rates 2050	0.024	0.024	0.024	0.025
Congo Brazzaville	Twinning rates 2050	0.030	0.030	0.029	0.031
Mali	Twinning rates 2050	0.033	0.033	0.031	0.035
Sierra Leone	Twinning rates 2050	0.035	0.035	0.033	0.037
Senegal	Twinning rates 2050	0.041	0.041	0.039	0.042
Cameroon	Twinning rates 2050	0.042	0.042	0.041	0.044
Angola	Twinning rates 2050	0.043	0.043	0.040	0.045
Gabon	Twinning rates 2050	0.043	0.043	0.041	0.045
Madagascar	Twinning rates 2050	0.043	0.043	0.041	0.046
Guinea	Twinning rates 2050	0.045	0.046	0.043	0.047
Central African Republic	Twinning rates 2050	0.047	0.047	0.043	0.051
Chad	Twinning rates 2050	0.049	0.049	0.046	0.053
Uganda	Twinning rates 2050	0.052	0.052	0.050	0.054
Lesotho	Twinning rates 2050	0.052	0.053	0.050	0.055
Pakistan	Twinning rates 2050	0.053	0.053	0.050	0.056
Comoros	Twinning rates 2050	0.062	0.062	0.059	0.065
Kenya	Twinning rates 2050	0.065	0.065	0.062	0.068
Rwanda	Twinning rates 2050	0.080	0.080	0.077	0.083
Burundi	Twinning rates 2050	0.082	0.082	0.078	0.085
Zimbabwe	Twinning rates 2050	0.084	0.085	0.081	0.087
Afghanistan	Twinning rates 2050	0.086	0.086	0.082	0.091
Ethiopia	Twinning rates 2050	0.088	0.088	0.084	0.093
Gambia	Twinning rates 2050	0.093	0.093	0.088	0.098
Namibia	Twinning rates 2050	0.098	0.098	0.094	0.103
Nepal	Twinning rates 2050	0.248	0.254	0.222	0.278
Bangladesh	Twinning rates 2050	0.309	0.313	0.284	0.336
Maldives	Twinning rates 2050	0.373	0.381	0.339	0.413
India	Twinning rates 2050	0.548	0.553	0.512	0.588
Togo	Twinning rates 2100	0.034	0.034	0.032	0.035
Benin	Twinning rates 2100	0.061	0.061	0.059	0.062
Ghana	Twinning rates 2100	0.071	0.071	0.069	0.073
Nigeria	Twinning rates 2100	0.080	0.080	0.077	0.083
Congo Brazzaville	Twinning rates 2100	0.083	0.083	0.080	0.086
Cote D'Ivoire	Twinning rates 2100	0.085	0.085	0.082	0.088
Senegal	Twinning rates 2100	0.087	0.087	0.084	0.090
Liberia	Twinning rates 2100	0.088	0.088	0.085	0.091
Burkina Faso	Twinning rates 2100	0.098	0.098	0.095	0.101
Comoros	Twinning rates 2100	0.100	0.101	0.096	0.105
Gabon	Twinning rates 2100	0.103	0.103	0.098	0.107
Malawi	Twinning rates 2100	0.104	0.104	0.101	0.107
Niger	Twinning rates 2100	0.111	0.111	0.106	0.116
Sierra Leone	Twinning rates 2100	0.111	0.111	0.107	0.116
Mozambique	Twinning rates 2100	0.112	0.112	0.108	0.116
Guinea	Twinning rates 2100	0.113	0.113	0.109	0.117
Zambia	Twinning rates 2100	0.113	0.113	0.109	0.117
Cameroon	Twinning rates 2100	0.114	0.114	0.110	0.117
Burundi	Twinning rates 2100	0.136	0.137	0.131	0.142
Rwanda	Twinning rates 2100	0.139	0.139	0.134	0.143
Mali	Twinning rates 2100	0.143	0.143	0.138	0.148
Gambia	Twinning rates 2100	0.148	0.149	0.141	0.156
Angola	Twinning rates 2100	0.151	0.152	0.144	0.160
Chad	Twinning rates 2100	0.153	0.153	0.146	0.159
Pakistan	Twinning rates 2100	0.158	0.159	0.151	0.165
Uganda	Twinning rates 2100	0.168	0.169	0.162	0.175
Kenya	Twinning rates 2100	0.169	0.169	0.163	0.175

(continued)

Supplementary Table S4. Continued

Country	Scenario	Median	Mean	Q25	Q75
Lesotho	Twinning rates 2100	0.179	0.179	0.171	0.187
Namibia	Twinning rates 2100	0.183	0.184	0.175	0.192
Ethiopia	Twinning rates 2100	0.186	0.186	0.178	0.194
Zimbabwe	Twinning rates 2100	0.191	0.191	0.184	0.198
Central African Republic	Twinning rates 2100	0.200	0.202	0.186	0.215
Madagascar	Twinning rates 2100	0.233	0.234	0.220	0.247
Afghanistan	Twinning rates 2100	0.244	0.244	0.237	0.251
Maldives	Twinning rates 2100	0.328	0.335	0.298	0.364
Bangladesh	Twinning rates 2100	0.653	0.661	0.605	0.708
India	Twinning rates 2100	0.752	0.759	0.703	0.807
Nepal	Twinning rates 2100	0.773	0.792	0.694	0.866
Maldives	Number of twin births in 2100	−0.390	−0.387	−0.404	−0.374
Lesotho	Number of twin births in 2100	−0.209	−0.208	−0.214	−0.203
Bangladesh	Number of twin births in 2100	−0.205	−0.202	−0.228	−0.179
Nepal	Number of twin births in 2100	−0.132	−0.122	−0.171	−0.086
India	Number of twin births in 2100	−0.111	−0.107	−0.135	−0.083
Sierra Leone	Number of twin births in 2100	−0.056	−0.056	−0.060	−0.053
Comoros	Number of twin births in 2100	−0.040	−0.040	−0.044	−0.036
Pakistan	Number of twin births in 2100	0.087	0.088	0.081	0.094
Kenya	Number of twin births in 2100	0.106	0.107	0.101	0.112
Zimbabwe	Number of twin births in 2100	0.180	0.181	0.173	0.187
Namibia	Number of twin births in 2100	0.192	0.193	0.184	0.201
Ghana	Number of twin births in 2100	0.208	0.209	0.206	0.211
Gambia	Number of twin births in 2100	0.221	0.222	0.214	0.230
Nigeria	Number of twin births in 2100	0.233	0.233	0.230	0.236
Guinea	Number of twin births in 2100	0.296	0.296	0.291	0.301
Liberia	Number of twin births in 2100	0.299	0.299	0.295	0.303
Gabon	Number of twin births in 2100	0.303	0.303	0.298	0.308
Rwanda	Number of twin births in 2100	0.328	0.329	0.323	0.334
Burkina Faso	Number of twin births in 2100	0.354	0.354	0.351	0.358
Ethiopia	Number of twin births in 2100	0.411	0.412	0.402	0.421
Malawi	Number of twin births in 2100	0.418	0.418	0.414	0.422
Uganda	Number of twin births in 2100	0.424	0.424	0.417	0.432
Burundi	Number of twin births in 2100	0.491	0.492	0.484	0.499
Afghanistan	Number of twin births in 2100	0.503	0.503	0.495	0.512
Congo Brazzaville	Number of twin births in 2100	0.628	0.628	0.623	0.632
Central African Republic	Number of twin births in 2100	0.639	0.641	0.620	0.660
Mozambique	Number of twin births in 2100	0.680	0.681	0.674	0.687
Madagascar	Number of twin births in 2100	0.751	0.753	0.733	0.771
Togo	Number of twin births in 2100	0.757	0.757	0.754	0.759
Zambia	Number of twin births in 2100	0.801	0.801	0.795	0.807
Cameroon	Number of twin births in 2100	0.856	0.856	0.851	0.862
Cote D'Ivoire	Number of twin births in 2100	0.885	0.885	0.880	0.890
Senegal	Number of twin births in 2100	1.028	1.029	1.023	1.034
Mali	Number of twin births in 2100	1.083	1.083	1.073	1.092
Chad	Number of twin births in 2100	1.086	1.086	1.074	1.098
Benin	Number of twin births in 2100	1.150	1.150	1.147	1.153
Angola	Number of twin births in 2100	1.182	1.183	1.168	1.198
Niger	Number of twin births in 2100	2.826	2.827	2.810	2.842

For each scenario, countries are ranked by the ascending order of mean percent changes.

© The Author(s) 2024. Published by Oxford University Press on behalf of European Society of Human Reproduction and Embryology.
This is an Open Access article distributed under the terms of the Creative Commons Attribution License (https://creativecommons.org/licenses/by/4.0/), which permits unrestricted reuse, distribution, and reproduction in any medium, provided the original work is properly cited.
Human Reproduction, 2024, 00, 1–19
https://doi.org/10.1093/humrep/deae276
Original Article
