# Supplementary material for: More twins expected in low-income countries with later maternal ages at birth and population growth
Source: Hum Reprod. 2024 Dec 26;40(2):372–81. doi: 10.1093/humrep/deae276 (PMC11788213; doi:10.1093/humrep/deae276)
Supplement: deae276_Supplementary_Table_S5 [file deae276_supplementary_table_s5.docx]

## Supplementary Table S5. Difference between the predicted percent changes in twinning rates and number of twin births, by different scenarios

Here we discuss in detail different sources of the projected changes in twinning rates and the number of twin births.

Increase in twinning rates and the number of twin births has different sources, namely the population size, age-specific fertility rate (ASFR), and age pattern of fertility, with mean age at childbearing expected in increase in most countries. These main factors can be broken down further into inter-related concepts:

A = Population Size, females (= Sum of the values in Age Structure [C])

B = Age Proportion (= Proportion of the values in Age Structure [C])

C = Age Structure

D = Age-specific fertility rate (ASFR)

E = Number of Births

F = Age Proportion of Births (= Proportion of the values in Number of Births [E])

<Example: Values for 2021 World population, from WPP>

|  | Age group | | | | | | | | |
| --- | --- | --- | --- | --- | --- | --- | --- | --- | --- |
|  | 10-14 | 15-19 | 20-24 | 25-29 | 30-34 | 35-39 | 40-44 | 45-49 | 50-54 |
| A | 2,481,279,000 | | | | | | | | |
| B | 0.13 | 0.12 | 0.12 | 0.12 | 0.12 | 0.11 | 0.10 | 0.10 | 0.09 |
| C | 319,110,000 | 301,699,000 | 290,940,000 | 288,912,000 | 296,544,.000 | 274,850,000 | 247,021,000 | 236,494,000 | 225,709,000 |
| D^1^ | 1.56 | 42.45 | 117.67 | 129.83 | 96.55 | 53.70 | 18.58 | 3.76 | 0.24 |
| E | 499,000 | 12,806,000 | 34,235,000 | 37,509,000 | 28,632,000 | 14,761,000 | 4590,000 | 888,000 | 55,000 |
| F | 0.004 | 0.096 | 0.256 | 0.280 | 0.214 | 0.110 | 0.034 | 0.007 | 0.000 |

^1^Number of births per 1,000 women

The projection of twinning rate and number of twin births is made based on E. With a given age-specific twinning probability, twinning rates will change only if the proportion within E (i.e., F) changes, and number of twin births will change only if the sum of E changes.

Since E is the product of C and D, any changes in C and/or D will result in changes in E and consequently twinning rates and number of twin births. Changes in D will only result in the change in twinning rates. Changes in C can come from different sources and with different consequences: Changes in C only through A will result in the change in the number of twin births, while changes in C only through B will make twinning rates different. Changes in C through both A and B will make both twinning rates and the number of twin births different.

To examine to what extent different sources of changes contribute to the change in twinning rates and number of twin births, we took the following steps:

First, for twinning rates, for each country we calculate twinning rates in two scenarios: i) Fix B (age proportion of reproductive-aged women) at year 2010 and only change D (ASFR) to the level projected for 2100; ii) Fix D at year 2010 and only change B to the level projected for 2100. We calculate percentage change in twinning rates compared to the baseline (twinning rates in year 2010) for each of the two scenarios, and then calculate percentage point difference between the percentage for the scenario i) from the scenario ii). We find that both scenarios make very similar amount of change in twinning rates (Figure S4, top). The percent point difference is mostly small, suggesting that the aging of maternal age at birth (B) and the shift of reproduction to older age (D) contribute almost equally to the expected change in twinning rates. The relatively larger percent point difference in India, Bangladesh, and Nepal means a relatively larger role played by the expected change in ASFR. Percent change values are shown in the table.

Second, for the number of twin births, for each country we calculate the number of twin births in two scenarios: i) Fix A (population size) at year 2010 and only change B (age proportion of reproductive-aged women) to the level projected for 2100; ii) Fix B at year 2010 and only change A to the level projected for 2100. Doing so acknowledges different contributions of population increase and the aging structure of women in reproductive age. Here, D is always fixed at year 2010. Similar to what we did for twinning rates, we calculate percent change in the number of twin births compared to the baseline (number of twin births in year 2010) for each scenario, and then calculate the point difference between the percentage for the scenario i) from the scenario ii). We find that (Figure S4, bottom) the number of twin births will be more influenced by the increasing population size than by the aging of maternal age structure. In contrast, percent points difference is very small (and slightly negative) in Maldives, Nepal, Bangladesh, and India. This suggests that the expected population decline in these countries by 2100 will play a slightly larger role in the declining number of twin births, than the age proportion of reproductive-aged women which is projected to be relatively unchanged compared to 2010. The latter contrasts with the projected increase in twinning rates in these countries, suggesting that the change in ASFR will be largely responsible for this increasing twinning rates.

|  | Twinning rates | | Number of twin births | |
| --- | --- | --- | --- | --- |
|  | *% Change due to age structure* | *% Change due to ASFR* | *% Change due to age proportion* | *% Change due to population size* |
| Afghanistan | 16.3 | 12.8 | 6.9 | 325.4 |
| Angola | 8.3 | 8.7 | 0.2 | 509.3 |
| Bangladesh | 11.5 | 56.4 | -10.5 | -19.6 |
| Benin | 3.5 | 3.1 | -2.7 | 425.1 |
| Burkina Faso | 7.1 | 4 | -2.2 | 304.8 |
| Burundi | 10.3 | 5.2 | 2.1 | 342 |
| Cameroon | 5.8 | 6.4 | -5.1 | 359.3 |
| Central African Republic | 12.6 | 10 | 3.2 | 344.3 |
| Chad | 7.7 | 10.1 | -0.4 | 541.3 |
| Comoros | 5.9 | 5.4 | -0.1 | 123.4 |
| Congo Brazzaville | 3.4 | 5.6 | -5.2 | 278.3 |
| Cote D'Ivoire | 4.5 | 4.9 | -4.7 | 349.3 |
| Ethiopia | 11.4 | 10.5 | 3.7 | 254.2 |
| Gabon | 5.2 | 6.3 | -6.3 | 189.7 |
| Gambia | 6.7 | 9.9 | 0.2 | 238.3 |
| Ghana | 4.5 | 3.3 | -2.8 | 162.4 |
| Guinea | 7.3 | 5.8 | -1.3 | 244.7 |
| India | 11.3 | 66.3 | -4.9 | -10.6 |
| Kenya | 8.5 | 10.6 | -4.7 | 153.1 |
| Lesotho | 9.9 | 9.5 | -5.5 | 38.1 |
| Liberia | 4.5 | 5 | -5.9 | 237.3 |
| Madagascar | 13 | 13.1 | 1.6 | 277.7 |
| Malawi | 7.5 | 4.2 | -7.4 | 307 |
| Maldives | 11.7 | 23.2 | -8.3 | -20.1 |
| Mali | 8.1 | 8.1 | -2.8 | 544.5 |
| Mozambique | 7 | 5 | -3.5 | 380.6 |
| Namibia | 9.1 | 11.6 | 0.5 | 112.5 |
| Nepal | 27.1 | 56.8 | 0.9 | -9.1 |
| Niger | 6.9 | 5.9 | 0.6 | 1121 |
| Nigeria | 5.7 | 3.6 | -1.6 | 267.1 |
| Pakistan | 11.2 | 6.7 | 1.2 | 137.9 |
| Rwanda | 8.8 | 6.1 | 1.6 | 200.9 |
| Senegal | 5.5 | 4.4 | -0.2 | 369.3 |
| Sierra Leone | 6.4 | 6.5 | -3.1 | 166.3 |
| Togo | 3.6 | 0.5 | -2.6 | 320.6 |
| Uganda | 11.6 | 7.5 | 0.6 | 337.4 |
| Zambia | 7.3 | 5.3 | -3.8 | 377.6 |
| Zimbabwe | 7.7 | 12.2 | -11.8 | 158.1 |
